# Supplementary material for: Modeling glioblastoma heterogeneity as a dynamic network of cell states
Source: Mol Syst Biol. 2021 Sep 16;17(9):e10105. doi: 10.15252/msb.202010105 (PMC8444284; doi:10.15252/msb.202010105)
Supplement: Supplementary file 6 — Source Data for Figure 5 [file MSB-17-e10105-s004.zip › Figure5A_sourcedata/GSEA_3017/hallmarks_stateF.GseaPreranked.1621934628066/HALLMARK_GLYCOLYSIS.html]

Details for gene set HALLMARK\_GLYCOLYSIS[GSEA]

|  || Dataset | state33017 |
| Phenotype | NoPhenotypeAvailable |
| Upregulated in class | na\_neg |
| GeneSet | HALLMARK\_GLYCOLYSIS |
| Enrichment Score (ES) | -0.15419996 |
| Normalized Enrichment Score (NES) | -0.8003187 |
| Nominal p-value | 0.7681818 |
| FDR q-value | 0.73477936 |
| FWER p-Value | 1.0 |
Table: GSEA Results Summary

  

Fig 1: Enrichment plot: HALLMARK\_GLYCOLYSIS      
 Profile of the Running ES Score & Positions of GeneSet Members on the Rank Ordered List

  

| PROBE | GENE SYMBOL | GENE\_TITLE | RANK IN GENE LIST | RANK METRIC SCORE | RUNNING ES | CORE ENRICHMENT || 1 | CHPF |  |  | 96 | 0.758 | -0.0049 | Yes |
| 2 | PLOD2 |  |  | 183 | 0.607 | -0.0130 | Yes |
| 3 | CD44 |  |  | 191 | 0.600 | 0.0143 | Yes |
| 4 | FUT8 |  |  | 194 | 0.593 | 0.0435 | Yes |
| 5 | DDIT4 |  |  | 216 | 0.579 | 0.0634 | Yes |
| 6 | RRAGD |  |  | 218 | 0.578 | 0.0923 | Yes |
| 7 | FAM162A |  |  | 282 | 0.524 | 0.0904 | Yes |
| 8 | PAM |  |  | 361 | 0.483 | 0.0797 | Yes |
| 9 | HSPA5 |  |  | 373 | 0.480 | 0.0991 | Yes |
| 10 | ALDOA |  |  | 377 | 0.479 | 0.1221 | Yes |
| 11 | VCAN |  |  | 410 | 0.466 | 0.1313 | Yes |
| 12 | GNPDA1 |  |  | 523 | 0.425 | 0.1022 | No |
| 13 | IRS2 |  |  | 595 | 0.400 | 0.0904 | No |
| 14 | SDC2 |  |  | 596 | 0.399 | 0.1107 | No |
| 15 | CHST2 |  |  | 628 | 0.390 | 0.1165 | No |
| 16 | VLDLR |  |  | 713 | 0.368 | 0.0972 | No |
| 17 | AGRN |  |  | 751 | 0.359 | 0.0987 | No |
| 18 | TPST1 |  |  | 767 | 0.354 | 0.1099 | No |
| 19 | VEGFA |  |  | 840 | 0.339 | 0.0945 | No |
| 20 | SDC3 |  |  | 863 | 0.333 | 0.1015 | No |
| 21 | MDH1 |  |  | 924 | 0.322 | 0.0907 | No |
| 22 | AGL |  |  | 976 | 0.314 | 0.0835 | No |
| 23 | CXCR4 |  |  | 1003 | 0.309 | 0.0875 | No |
| 24 | B3GAT1 |  |  | 1145 | 0.286 | 0.0382 | No |
| 25 | GPC1 |  |  | 1162 | 0.284 | 0.0454 | No |
| 26 | CHST12 |  |  | 1248 | 0.274 | 0.0209 | No |
| 27 | ALDH7A1 |  |  | 1255 | 0.273 | 0.0321 | No |
| 28 | CAPN5 |  |  | 1339 | 0.263 | 0.0079 | No |
| 29 | CITED2 |  |  | 1433 | 0.254 | -0.0213 | No |
| 30 | SDC1 |  |  | 1438 | 0.254 | -0.0102 | No |
| 31 | ENO1 |  |  | 1673 | -0.308 | -0.1004 | No |
| 32 | UGP2 |  |  | 1725 | -0.327 | -0.1068 | No |
| 33 | KIF20A |  |  | 1792 | -0.358 | -0.1185 | No |
| 34 | SAP30 |  |  | 1826 | -0.378 | -0.1142 | No |
| 35 | LDHA |  |  | 1833 | -0.380 | -0.0976 | No |
| 36 | FKBP4 |  |  | 1903 | -0.426 | -0.1072 | No |
| 37 | NASP |  |  | 1988 | -0.481 | -0.1207 | No |
| 38 | STMN1 |  |  | 2063 | -0.567 | -0.1254 | No |
| 39 | CENPA |  |  | 2073 | -0.586 | -0.0997 | No |
| 40 | AURKA |  |  | 2110 | -0.673 | -0.0818 | No |
| 41 | DEPDC1 |  |  | 2152 | -0.809 | -0.0593 | No |
| 42 | HMMR |  |  | 2179 | -0.895 | -0.0256 | No |
| 43 | CDK1 |  |  | 2225 | -1.153 | 0.0127 | No |
Table: GSEA details [plain text format]

  

Fig 2: HALLMARK\_GLYCOLYSIS: Random ES distribution      
 Gene set null distribution of ES for **HALLMARK\_GLYCOLYSIS**
